# Walking Is Not Like Reaching: Evidence from Periodic Mechanical Perturbations

**DOI:** 10.1371/journal.pone.0031767

**Published:** 2012-03-27

**Authors:** Jooeun Ahn, Neville Hogan

**Affiliations:** 1 Department of Mechanical Engineering, Massachusetts Institute of Technology, Cambridge, Massachusetts, United States of America; 2 Department of Brain and Cognitive Sciences, Massachusetts Institute of Technology, Cambridge, Massachusetts, United States of America; The University of Western Ontario, Canada

## Abstract

The control architecture underlying human reaching has been established, at least in broad outline. However, despite extensive research, the control architecture underlying human locomotion remains unclear. Some studies show evidence of high-level control focused on lower-limb trajectories; others suggest that nonlinear oscillators such as lower-level rhythmic central pattern generators (CPGs) play a significant role. To resolve this ambiguity, we reasoned that if a nonlinear oscillator contributes to locomotor control, human walking should exhibit *dynamic entrainment* to periodic mechanical perturbation; entrainment is a distinctive behavior of nonlinear oscillators. Here we present the first behavioral evidence that nonlinear neuro-mechanical oscillators contribute to the production of human walking, albeit weakly. As unimpaired human subjects walked at constant speed, we applied periodic torque pulses to the ankle at periods different from their preferred cadence. The gait period of 18 out of 19 subjects entrained to this mechanical perturbation, converging to match that of the perturbation. Significantly, entrainment occurred only if the perturbation period was close to subjects' preferred walking cadence: it exhibited a *narrow basin of entrainment*. Further, regardless of the phase within the walking cycle at which perturbation was initiated, subjects' gait synchronized or *phase-locked* with the mechanical perturbation at a phase of gait where it assisted propulsion. These results were affected neither by auditory feedback nor by a distractor task. However, the convergence to phase-locking was slow. These characteristics indicate that nonlinear neuro-mechanical oscillators make at most a modest contribution to human walking. Our results suggest that human locomotor control is not organized as in reaching to meet a predominantly kinematic specification, but is hierarchically organized with a semi-autonomous peripheral oscillator operating under episodic supervisory control.

## Introduction

Despite extensive research, the control of human locomotion remains unclear. Walking in unimpaired adults is characterized by a remarkably repeatable spatial trajectory of the foot [1]. In response to surface irregularity in the form of small obstacles, subjects adjusted their minimum toe clearance using subtle adjustments of lower-limb kinematics [2]. Patients with spinal cord injury (SCI) who recovered following body-weight supported treadmill training generated a foot trajectory that closely matched the normal pattern, although they used very different joint coordination patterns to do so [3]. These observations suggest that supra-spinal processes predominate, adjusting peripheral muscle activation and joint recruitment to control the kinematics of the foot. Discrete reaching with the hand is similar: horizontal-plane hand paths are predominantly straight and hand speed profiles are remarkably invariant to reaching direction, load carried and movement speed [4], [5]. Following exposure to mechanical perturbations, subjects adapt largely to restore hand kinematics [6], [7]. Following exposure to visual distortions, subjects adapt largely to restore the visual appearance of the controlled-point (cursor) kinematics [8], [9]. These and many other studies indicate that muscle activations are adjusted as needed to meet a centrally-planned kinematic specification.

Insofar as the foot is the lower-limb “end-effector”, loosely analogous to the hand, the control architecture for locomotion appears similar to that observed in upper-limb reaching. However, locomotion is a predominantly rhythmic activity and neural control of rhythmic movement is substantially different from discrete reaching. Rhythmic behavior is very old phylogenetically and available evidence indicates that oscillations are a primitive element of biological motor control. The relation between discrete and rhythmic movements has been studied extensively [10], [11], [12]. Unimpaired humans executing discrete movements activate substantially more brain regions than when they execute rhythmic movements [13]. The difference impacts the acquisition of skilled behavior: learning eye-hand coordination to compensate for visual field distortion is slower for rhythmic movements and transfers poorly to discrete reaching, whereas adaptation learned from discrete reaches transfers well to rhythmic movements [14]. These observations suggest that the control of rhythmic actions may be situated deeper in the central nervous system (CNS) perhaps with prominent contributions from the spinal cord. Observations of fictive locomotion in non-human vertebrates provide unequivocal evidence that neural circuits capable of generating sustained rhythmic activity exist in the spinal cord isolated from its periphery, though sensory feedback is known to play a key role [15], [16], [17], [18], [19], [20], [21]. For unimpaired humans, continuous leg muscle vibration produced locomotor-like stepping movements, and spinal electromagnetic stimulation applied to unimpaired human vertebrae induced involuntary locomotor-like movements [22], [23]. That suggests the existence of a rhythmic central pattern generator (CPG) in the human spinal cord that may contribute to generating locomotor activity, though feedback related to limb loading, hip extension or the skin of the foot also play important roles [16], [24], [25].

The relative contribution of rhythmic pattern generation to unimpaired human locomotion remains unclear. Human infants exhibit a primitive rhythmic stepping reflex but it typically disappears at about 6 weeks after birth without training [26]. When independent walking emerges at about a year old, it does not initially exhibit the rhythmic pattern of mature walking and this cannot be ascribed to immature postural control [27]. The locomotor-like movements evoked by stimuli to unimpaired human subjects were observed in a gravity-neutral position, unlike normal walking, rendering it difficult to assess how those results would apply to upright walking [22], [23]. In this paper we report behavioral experiments with unimpaired human subjects that attempted to (1) test whether a neuro-mechanical oscillator contributes to level walking and (2) assess the strength of its contribution.

Robustly sustained oscillation can only emerge from a nonlinear dynamical system. While a linear spring interacting with a mass without friction (the classic simple harmonic oscillator) exhibits oscillatory behavior, it is neither stable (the system will not return to its original oscillation after perturbation) nor robust (infinitesimal changes in friction will prevent sustained oscillation). Robustly sustained oscillation emerges as a *limit cycle attractor* from nonlinear dynamical systems such as relaxation oscillators [28]. Nonlinear limit cycle oscillators not only encapsulate the robust and stable rhythmic motion of periphery in human walking; they also serve as competent models of neural rhythmic pattern generators [29], [30], [31]. One of their distinctive characteristics is that they may exhibit *dynamic entrainment* (an observation credited to Christiaan Huygens in 1665): under certain conditions they will synchronize their period of oscillation to that of an imposed perturbation, *phase-locking* to establish a particular phase relation with it [32]. Usually entrainment occurs only for a limited range of perturbation frequencies; it exhibits a *finite basin of entrainment*. In fact, entrainment to periodic mechanical perturbation has been reported in several non-human vertebrates which show clear evidence of spinal pattern generators [17], [33], [34], [35].

We reasoned that if a nonlinear limit-cycle oscillator plays a significant role in normal human locomotion, entrainment to periodic mechanical perturbation should be observable. Conversely, if human locomotion is predominantly controlled to meet a centrally-specified time course of kinematics (such as the trajectory of the foot) then entrainment to mechanical perturbation should not be observed under modest mechanical perturbation; human walking should try to preserve the specified kinematic patterns (including the specified walking cadence) instead of allowing synchrony to external mechanical perturbation, i.e., entrainment. In this study, we perturbed treadmill walking of unimpaired subjects by applying modest periodic plantar-flexion torques to the ankle using a robotic device. Synchronization occurred with a finite basin of entrainment, robustly phase-locking to the perturbation such that it assisted propulsion, demonstrating the presence of a nonlinear neuro-mechanical oscillator.

## Methods

### Ethics Statement

Nineteen young adult subjects participated in the study (ages 23 to 35). They all reported no neurological or biomechanical impairment. They walked on a treadmill at a self-selected comfortable speed while Anklebot, a wearable robot, applied a program of mechanical perturbations.

The Committee on the Use of Humans as Experimental Subjects (COUHES), which acts as the Institutional Review Board (IRB) for the Massachusetts Institute of Technology (MIT), specifically approved this study and all the subjects gave written informed consent to participate as approved by the committee.

### Equipment

**Figure 1:**
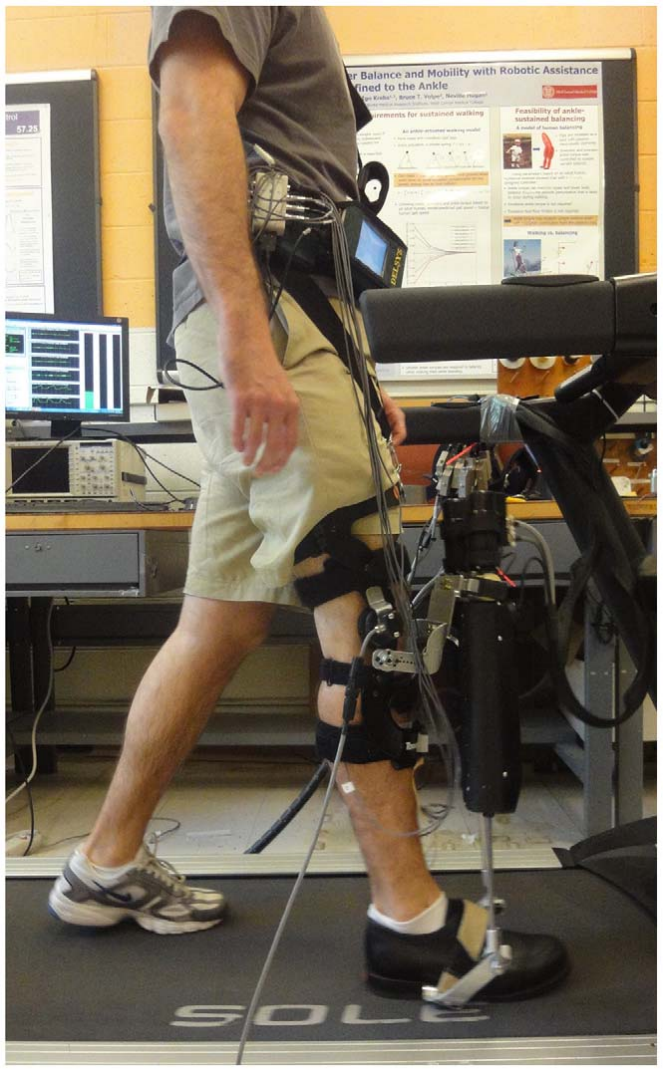
An unimpaired human subject wearing Anklebot while walking on a treadmill.

Anklebot (Interactive Motion Technologies, Inc.) (Figure 1) is a therapeutic robot designed to assist and evaluate ankle function [36]. It can deliver torque simultaneously in both dorsi/plantar-flexion and inversion/eversion, though in this study, we focused on the sagittal plane. The time profile of ankle torque actuation was programmed at a sampling rate of 200 Hz with a precision ≤2.82 N-m. Onboard sensors measured ankle angles in both dorsi/plantar-flexion and inversion/eversion with precision ≤1.5 degrees. A treadmill (Sole Fitness F80 with a 0.84 m×1.90 m deck and 0.1 mph (0.045 m/s) belt speed resolution) was used. In some experiments, subjects wore acoustic noise-cancelling headphones (Bose QuietComfort 3).

### Experimental Protocols

#### Experiment 1: Entrainment

To compensate for its possible effect on walking dynamics, throughout all trials Anklebot was programmed to act like a torsional spring and damper with constant equilibrium position, stiffness and damping. The equilibrium position was determined as the ankle angle when the subject stood upright. The stiffness was set as 5 N-m/rad, selected to approximate the stiffness necessary to compensate for the effect of Anklebot's inertia on the natural frequency of the body about the ankle1. The damping was chosen empirically to be 1 N-m-sec/rad; minimal to avoid impeding walking yet sufficient to stabilize Anklebot.

Before applying periodic mechanical perturbations, each subject's preferred stride duration was measured. Subjects were instructed to walk on the treadmill at their preferred gait cadence while wearing Anklebot on one leg. Each subject adjusted the speed of the treadmill to be comfortable for walking, and this treadmill speed was maintained throughout the subsequent experimental session. Each subject's preferred stride duration (τ_0_) was measured as the average duration of 15 successive strides.

After measuring τ_0_, periodic square torque pulses of magnitude 10 N-m and duration 0.1 second were added to the torque due to the programmed spring-damper behavior. A magnitude of 10 N-m is comparable to 10% of maximum ankle torque during normal walking in adults and 0.1 second is comparable to 10% of one stride duration in normal walking [39], [40], [41]. Subjects were instructed to continue walking until asked to stop. A trial began with a subject walking at preferred speed for at least 20 strides without perturbation. Then the experimenter initiated the periodic perturbation approximately coincident with the push-off portion of stance phase of the leg wearing Anklebot. Under computer control, Anklebot subsequently generated 30, 40 or 50 torque pulses at intervals of τ_P_. Thereafter the torque pulses were discontinued (but the spring-damper behavior was maintained). Subjects continued walking for at least 20 strides before being instructed to stop.

On different trials the period of perturbation (τ_P_) varied from lower to higher than τ_0_, discretized with a resolution of 50 ms. The subject's preferred stride duration was rounded to the nearest 50 ms and adjusted by adding and subtracting 50 ms and 100 ms respectively to determine four initial perturbation periods. An additional 20 ms was added by the Anklebot controller. Thus for a subject with a preferred stride duration of 1416 ms, we determined initial perturbations with periods of 1320 ms, 1370 ms, 1470 ms and 1520 ms. These were applied in random order. If the first three perturbations showed clear evidence of a finite basin of entrainment (defined in Data Analysis) the fourth was omitted and the session ended. If four perturbations did not show clear evidence of a finite basin of entrainment, further perturbation periods were added. If subjects exhibited entrainment to the shortest (longest) perturbation period, on subsequent trials we further reduced (increased) the period in steps of 50 ms until entrainment was no longer observed. All 19 subjects participated in this experiment. For 2 subjects, τ_P_ was discretized with 100 ms intervals; for 5 subjects, τ_P_ was discretized with 25 ms intervals.

#### Experiment 2: Transient Phase Dynamics

To investigate the transient process by which entrainment and phase-locking were achieved, we revised the protocol as follows. A perturbation period was selected which had evoked entrainment in experiment 1. As before, subjects walked at preferred speed for at least 20 strides without perturbation. Then the experimenter initiated the periodic perturbation, taking care that initiation occurred at one of a wide range of gait phases significantly different from the push-off portion of the stance phase of the leg wearing Anklebot. Under computer control, Anklebot subsequently generated 80 to 100 torque pulses at intervals of τ_P_. Thereafter the torque pulses were discontinued (but the spring-damper behavior was maintained). Subjects continued walking for at least 20 strides before being instructed to stop. Seven of the 19 subjects participated in this experiment.

#### Role of Auditory Feedback

When producing the square torque pulse, Anklebot made a small but perceptible noise. To assess and minimize the possible effect of auditory input on entrainment, 6 of the above 7 subjects were instructed to wear noise-cancelling headphones through which white noise was played during experiments 1 and 2. The volume of white noise was increased until subjects were unable to detect the noise made by Anklebot.

#### Role of Voluntary Intervention

To assess and minimize the likelihood of voluntary adjustment to the perturbation, 4 of the above 6 subjects (who wore noise-cancelling headphones) were asked to perform a distracting task, counting aloud backwards from 100 to 1 in their second language during experiments 1 and 2.

### Data Analysis

The torque profile exerted by Anklebot and the kinematics of the ankle and knee wearing the device were recorded at a sampling rate of 200 Hz using the onboard sensors. A gait cycle was defined from the knee angle data. Stride duration was compared before, during and after the perturbation in each trial. All statistical analysis was performed at a significance level of 5%.

#### Gait Cycle

**Figure 2:**
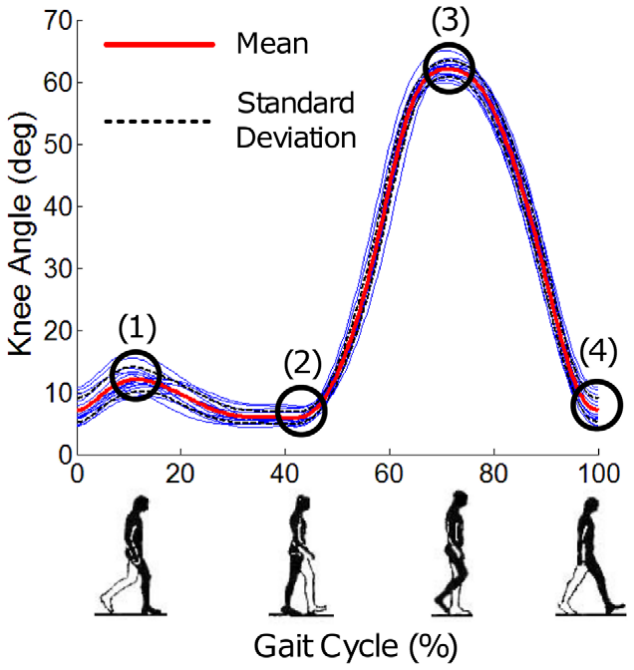
Typical plot of knee angle as measured by Anklebot vs. phase (% of gait cycle). The miniature icons of a walker illustrate the corresponding phases of a gait cycle. Four extrema were identified from zero crossings of the knee angular velocity: (1) maximum knee flexion during stance phase, (2) maximum knee extension during terminal stance phase, (3) maximum knee flexion during swing phase and (4) maximum knee extension adjacent to heel strike.

The knee angle profile was filtered with a digital low-pass filter with 7 Hz cutoff frequency. Key landmarks in the gait cycle were estimated from extrema of the filtered knee angle profile: (1) maximum stance phase knee flexion, (2) maximum knee extension in terminal stance phase, (3) maximum swing phase knee flexion and (4) maximum knee extension adjacent to heel strike (Figure 2). For each stride, the knee angle profile was normalized to define a gait phase running from 0 to 100% with 0% identified as the moment of local maximum knee extension (4) following maximum swing phase knee flexion (3), based on the observation that (4) is adjacent to the initial loading or heel strike in normal walking [39], [41]. For 2 subjects this proved unreliable and 0% was alternatively identified as the moment of maximum swing phase knee flexion (3) shifted by 74 (or −26)%, based on the observation that (3) occurs near 74% of gait cycle in normal walking [39], [41].

#### Assessment of Entrainment

**Figure 3:**
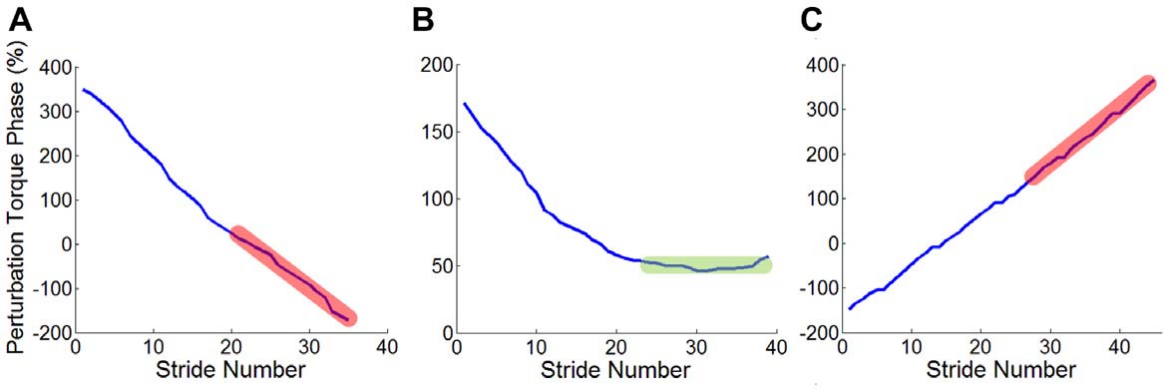
Phase of perturbation torque pulse vs. stride number plotted for three different trials. Entrainment was determined by linear regression of phase of perturbation onto stride number for the last 15 strides during perturbation: in A, the regression slope is significantly negative—a non-entrained gait with a fast perturbation; in B, the regression slope is not significantly different from zero—an entrained gait; in C, the regression slope is significantly positive—a non-entrained gait with a slow perturbation.

The torque perturbation was delivered at constant period throughout any one trial but the gait phase at which it occurred could vary (Figure 3). Entrainment requires gait to have the same period as the perturbation, which requires the perturbation phase to be independent of stride number. Entrainment was assessed by linear regression of phase with respect to stride number over the last 15 strides when perturbation was present and testing whether the slope was zero. A significant positive slope was classified as not entrained to a “slow” perturbation; a significant negative slope was classified as not entrained to a “fast” perturbation.

#### Basin of Entrainment

The basin of entrainment was estimated from the highest and lowest perturbation periods that entrained gait. Its upper boundary was assigned to the mid-point between the largest perturbation period that entrained gait and the smallest perturbation period identified as a slow perturbation that did not entrain gait. Its lower boundary was defined similarly.

#### Analysis of Phase Convergence

From the data of experiment 1 for each subject, the standard deviation, σ, of the gait phases at which the torque pulse occurred in the last 15 strides during perturbation of all entrained trials was determined. The distribution of these phases appeared Gaussian hence about 95% of them were expected to lie within an interval ±2σ wide. In experiment 2, the onset of phase-locking was determined by plotting the gait phase at which the torque pulse occurred as a function of stride number. A converged phase value, ϕ_converged_, was found which made an interval ϕ_converged_ ±2σ contain the greatest number of strides. In finding ϕ_converged_, some strides were allowed to exceed this range provided that no more than 5% of the total number of perturbed strides did so in succession. The onset of phase locking was determined as the first stride within the interval ϕ_converged_ ±2σ.

#### After-Effect

If entrainment occurred, possible after-effects of the perturbation were assessed by comparing the durations of 15 successive strides immediately before the beginning of perturbation with 15 successive strides immediately after the end of perturbation. An after-effect was defined as a statistically significant difference between stride duration before and after perturbation.

## Results

### Entrainment

**Figure 4:**
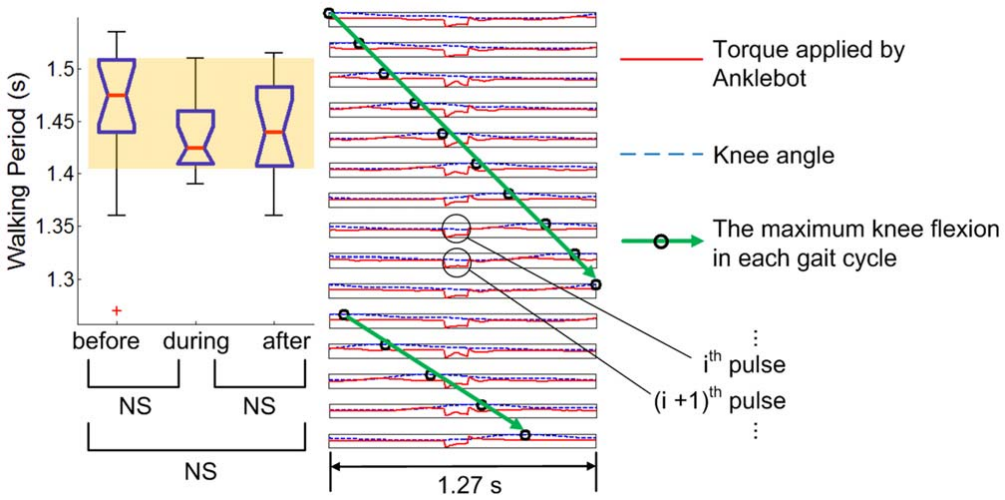
Typical results of a gait that did not entrain to “fast” perturbation (τ_P_<τ_0_). The box plot shows the distribution of walking periods of the last 15 strides before perturbation, the last 15 strides during perturbation and the first 15 strides after the end of perturbation. The knee angle and the torque pulse imposed by Anklebot during the last 15 perturbation periods are plotted next to the box plot; each row indicates knee angle (the dotted blue curve) and Anklebot torque profile (the solid red curve) during one perturbation cycle. For each cycle, the phase of maximum knee flexion is identified (the black circle) and the trend of the maximum knee flexion phase is visualized by a green arrow. Stride duration (shown in the box plot) did not change significantly due to the mechanical perturbations, and the phase of maximum knee flexion drifted continuously relative to the perturbation.

**Figure 5:**
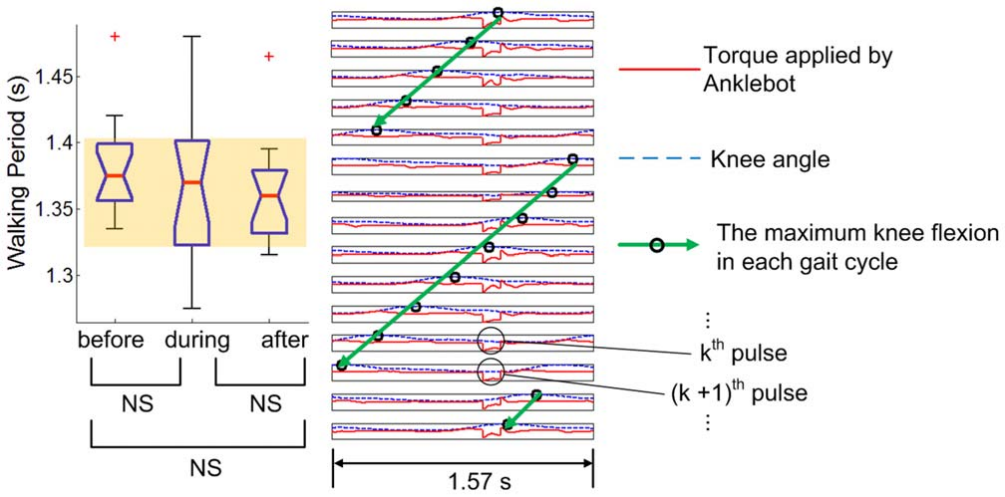
Typical results of a gait that did not entrain to “slow” perturbation (τ_P_>τ_0_). Stride duration (shown in the box plot) did not change significantly due to the mechanical perturbations, and the phase of maximum knee flexion drifted continuously relative to the perturbation. The direction of drift is opposite to Figure 4.

**Figure 6:**
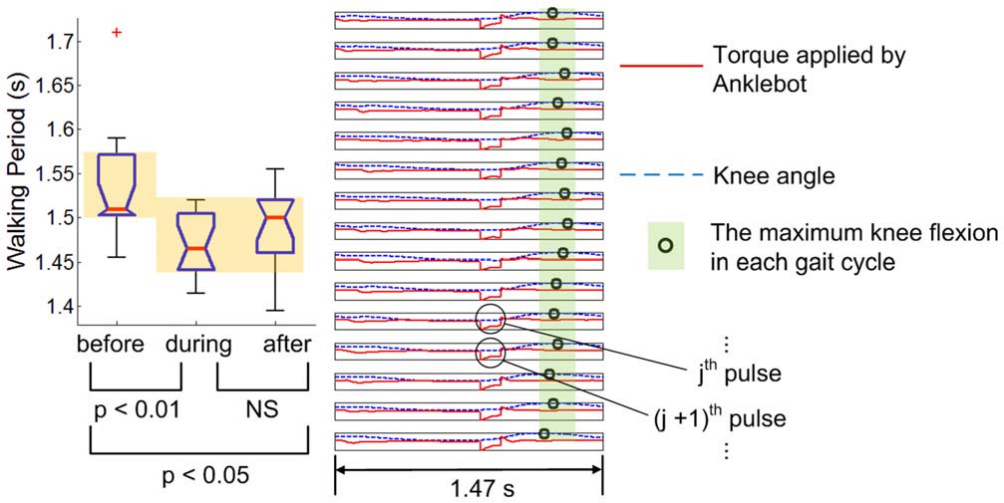
Typical results of a gait that entrained to perturbation. Stride duration (shown in the box plot) approximated τ_P_ with a statistically significant difference from the walking period before perturbation. The subject's cadence changed from the originally preferred value to synchronize with the periodic perturbation. Maximum knee flexion maintained a constant phase difference from the perturbation pulse instead of drifting relative to the perturbation pulse.

Entrainment was observed in 18 of 19 subjects when the perturbation period, τ_P_, was sufficiently close to the preferred stride period, τ_0_. Typical results are presented in Figure 4, Figure 5, and Figure 6.

#### Before Perturbation

Table 1 shows preferred treadmill walking speed, stride duration (mean, standard deviation and coefficient of variation) and stride length (normalized by subject height) of 15 successive strides immediately before the beginning of perturbation individually for each subject and averaged over all subjects. The values observed were comparable to those typically reported for slow overground walking [42].

**Table 1:** Subjects' preferred speeds, walking periods and normalized stride lengths.

		Walking period before perturbation (s)			
Subject ID (Gender)	Preferred treadmill speed (m/s)	Mean	SD	CV %	Stride length to height ratio %
1 (M)	0.99	1.44	0.074	5.1	80
2 (M)	0.81	1.40	0.025	1.8	65
3 (M)	0.81	1.20	0.038	3.2	53
4 (M)	0.86	1.31	0.063	4.8	62
5 (M)	0.99	1.20	0.020	1.7	65
6 (M)	0.81	1.54	0.059	3.8	69
7 (M)	0.99	1.28	0.031	2.4	71
8 (M)	0.95	1.21	0.033	2.7	63
9 (M)	0.90	1.24	0.031	2.5	63
10 (F)	0.90	1.22	0.030	2.5	66
11 (M)	0.99	1.33	0.033	2.5	74
12 (M)	0.86	1.45	0.043	2.9	68
13 (M)	0.99	1.23	0.026	2.1	67
14 (M)	0.95	1.32	0.048	3.6	70
15 (M)	0.77	1.64	0.037	2.3	72
16 (M)	0.77	1.67	0.049	2.9	73
17 (M)	0.9	1.47	0.078	5.3	73
18 (M)	0.77	1.50	0.071	4.7	62
19 (M)	0.99	1.19	0.028	2.3	71
**All subjects**	Mean = 0.89	1.38	0.16	Mean = 3.12	Mean = 68
	SD = 0.086			SD = 1.14	SD = 5.9

SD: standard deviation; CV: coefficient of variation (standard deviation/mean).

#### During Perturbation

If the period of perturbation, τ_P_, was sufficiently short (Figure 4) or long (Figure 5) entrainment was not observed. Maximum knee flexion, which should occupy an almost constant phase of the gait cycle, varied continuously with respect to the perturbation. In all, statistical analysis identified 46 trials out of 80 as not entrained. In 27 of those 46 trials, no significant difference between stride duration before and during perturbation was observed, indicating that the perturbation had little influence on gait in these cases.

Conversely, if the perturbation period was sufficiently close to the preferred stride period, τ_0_, entrainment was observed (Figure 6). Subjects' gait adapted so that the phase at which the imposed torque pulse occurred converged to a constant phase of the gait cycle. Statistical analysis identified entrainment in 18 of 19 subjects in one or more trials, and in 34 out of 80 trials overall. Due to the variability of normal walking, the pre-perturbation stride period often deviated from the preferred period. Therefore, although entrainment implied that the stride period converged to the perturbation period, which always differed from preferred stride period, entrainment was not always accompanied by a significant difference between stride duration before and during perturbation. However, a significant difference was observed for 20 of the 34 entrained trials.

#### Finite Basin of Entrainment

The observation that entrainment only occurred if the perturbation period was sufficiently similar to the preferred stride duration indicates a *finite basin of entrainment*. Table 2 shows the basin of entrainment of each subject expressed as a percentage of the subject's average walking period before perturbation, τ_before_. To compare it with the normal variability of walking, Table 2 also shows the basin of entrainment expressed as a percentage of a range containing 95% of the observed stride durations, four times the standard deviation of the subject's walking period before perturbation, σ_before_. The mean basin of entrainment was 6.7% of the pre-perturbation walking period and 56% of its four-sigma range. Only one subject (#11) out of 18 exhibited a basin of entrainment wider than the variability of pre-perturbation walking, and then only by 10%.

**Table 2:** Basin of entrainment normalized by walking cadence and its variability.

Subject ID	Number of trials	Basin of entrainment to walking cadence ratio %	Basin of entrainment to variability ratio %
1	4	7.0	34
2	4	7.1	100
3	4	4.2	33
4	5	3.8	20
5	5	4.2	62
6	5	3.1	20
7	3	3.9	40
8	3	4.1	38
9	5	8.1	79
10	3	2.1	21
11	5	11	110
12	4	6.9	59
13	3	2.0	24
14	3	7.6	52
15	5	9.2	100
16	5	9.0	77
17	4	14	64
18	6	13	71
19	4	N/A	N/A
**All subjects**	80	Mean = 6.7 SD = 3.6	Mean = 56 SD = 30

The basin of entrainment was compared with the average walking period and the variability of walking cadence. Basin of entrainment to walking cadence ratio was evaluated as 
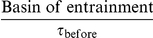
, where τ_before_ is the average walking period of last 15 strides before perturbation. Basin of entrainment to variability ratio was evaluated as 
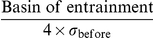
, where σ_before_ is the standard deviation of walking period of last 15 strides before perturbation. SD means standard deviation.

#### Phase-Locking

**Figure 7:**
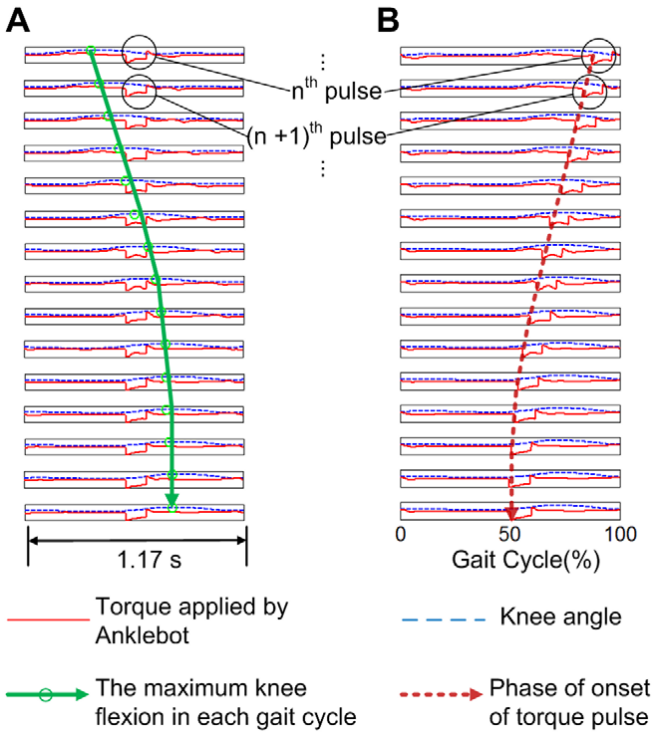
Transient behavior under perturbation. A shows the knee angle (the dotted blue curve), Anklebot torque profile (the solid red curve), and maximum knee flexion phase (the green circle and arrow) during the last 15 *perturbation cycles*, and B shows knee angle and Anklebot torque profile with the onset of torque pulse marked (the dotted brown arrow) during the last 15 *gait cycles* under perturbation. In A, the maximum knee flexion which should occupy an almost constant phase of gait cycle drifted initially but converged on a specific phase of the perturbation cycle. The convergence is also shown in B; the onset of torque pulse drifted initially, but converged on a specific phase of the gait cycle, which is close to 50%.

**Figure 8:**
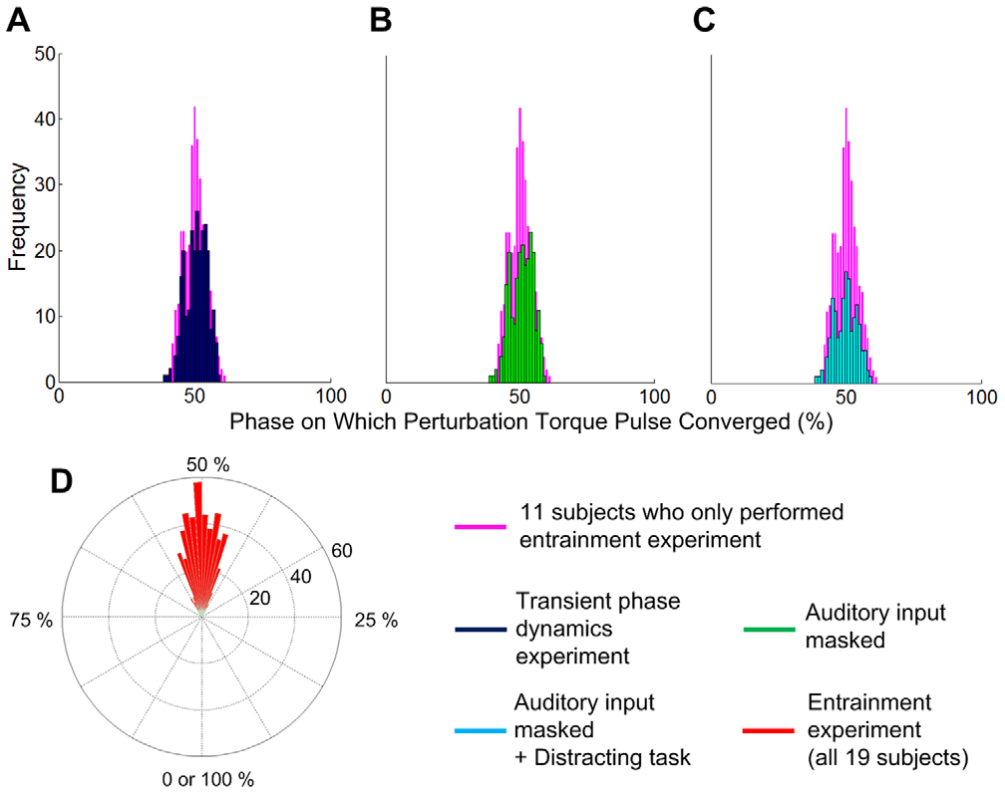
Histograms of the phase, ϕ_P_ at which the perturbation torque pulses occurred in phase-locked strides. Purple bars in A, B and C show the distribution of ϕ_P_ in the last 15 strides of all entrained trials of 11 subjects who only performed experiment 1. Superimposed on this histogram in A is the distribution of ϕ_P_ of phase-locked strides in experiment 2 (dark blue bars), in B with auditory input masked (green bars), and in C with auditory input masked and a distracting task (light blue bars). A polar (“rose”) plot of the histogram of all entrained trails of all 19 subjects in experiment 1 is shown in D showing that the distribution occupied a narrow region of the gait cycle. Statistical analysis indicated no significant difference between these distributions in mean or standard deviation.

In principle, entrainment only requires a subject's stride duration to converge to the period of the perturbation; convergence may occur with any phase relation between perturbation and entrained gait. That is, the relative phase difference between gait and perturbation must converge to a constant, but it may be any constant. Remarkably, we observed that, when gait was entrained, synchrony occurred at a specific phase. This is termed *phase-locking*. Figure 7 shows the transient behavior of one subject who was plausibly in the process of becoming entrained. The phase on which the perturbation torque pulse converged is close to 50% of the gait cycle. This is near the boundary between the terminal stance and pre-swing phases, and coincides approximately with maximum ankle actuation in normal human walking [39]. Figure 8 shows a histogram of the phase in gait cycle, ϕ_P_ at which the perturbation torque pulses occurred in the last 15 strides during perturbation of entrained gaits. The average and standard deviation of ϕ_P_ were 50.2 and 3.80 (%) respectively. This narrow distribution justified the use of standard statistical tests based on a Gaussian distribution.

#### After Perturbation

**Figure 9:**
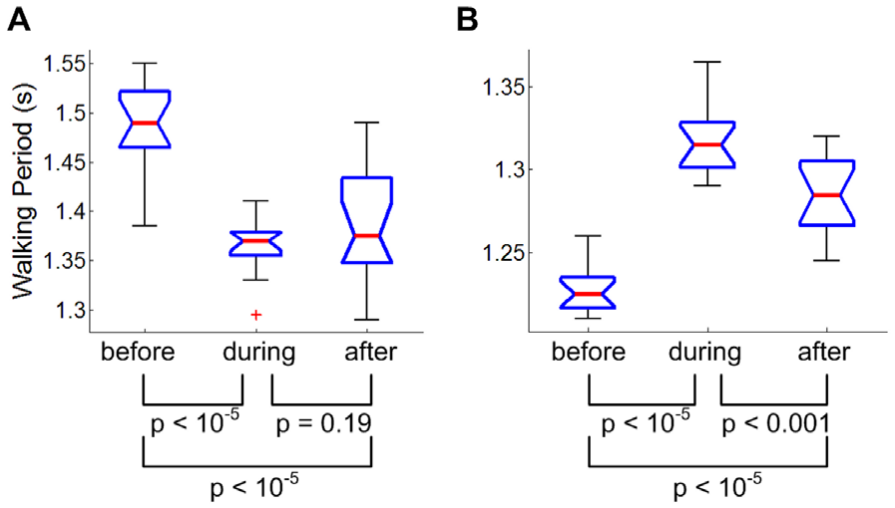
Two types of aftereffect. In A, there is significant difference between stride duration before and during perturbation, but no significant difference between during and after perturbation; in B, there is significant difference between stride duration before, during and after perturbation. For all trials classified into B, the mean stride duration after perturbation lay between its during-perturbation and pre-perturbation values.

An after-effect, which was defined as a statistically significant difference between stride duration before and after perturbation, was detected in 11 out of 18 subjects who showed entrainment. Nine of those 11 continued walking at the perturbation period, with no significant difference between stride duration during and after perturbation (as in Figure 6). Of the 20 entrained trials with a significant difference between stride duration before and during perturbation, 16 exhibited an after-effect. In 12 of those 16 trials, walking continued at the perturbation period with no significant difference between stride duration during and after perturbation (as in Figure 6). In the 4 remaining trials (from 2 subjects) the mean stride duration after perturbation was significantly different from its value during perturbation but lay between its values during and before perturbation. Representative data for each case are shown in Figure 9.

### Transient Phase Dynamics

**Figure 10:**
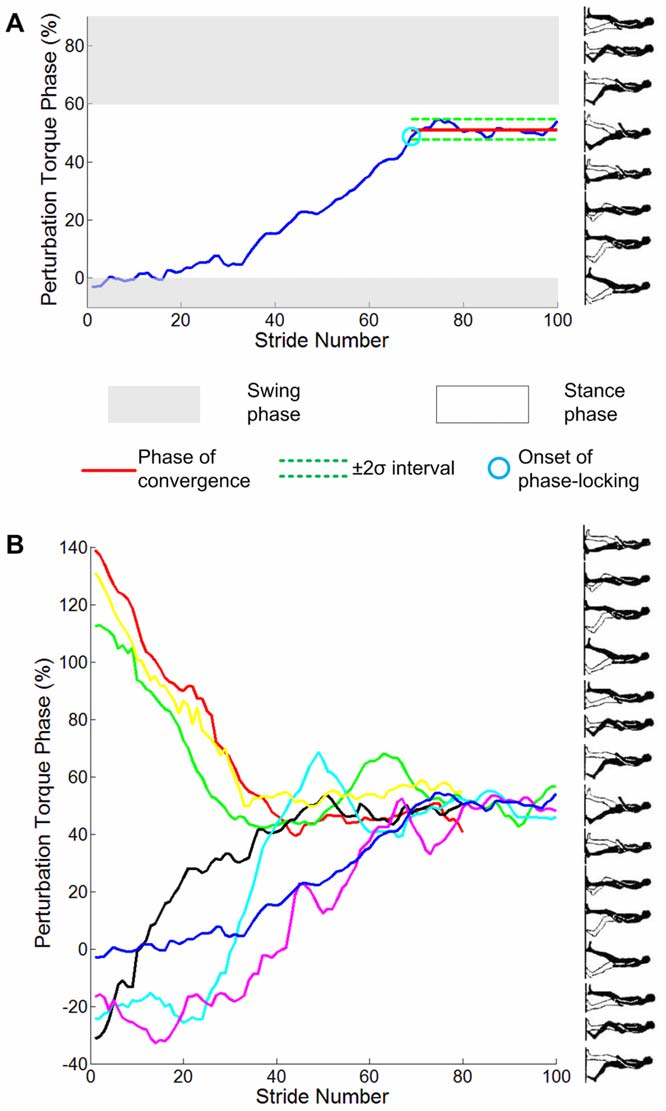
Phase of perturbation torque pulse vs. stride number in experiment 2. A illustrates typical phase locking of one subject; σ is the standard deviation of the gait phases at which the torque pulse occurred in the last 15 strides during perturbation of all entrained trials of the subject in experiment 1. The phase of convergence and onset of phase locking were determined as explained in Data Analysis. The miniature icons of a walker illustrate the corresponding phases of a gait cycle. The initial perturbation pulse was applied just before the beginning of a double stance phase (−5% gait cycle). Over 70 subsequent strides (140 steps) taking approximately 100 seconds, the subject gradually changed cadence to phase lock with the perturbation at 50% gait cycle, approximately the maximum ankle-actuation phase of normal human walking. B shows the phase locking of all 7 subjects who participated in experiment 2.

To test whether phase-locking was an artifact of initiating the periodic perturbation at the same approximate phase of gait, we performed experiment 2, initiating the periodic mechanical perturbation at phases far from ankle push-off. All seven subjects who participated in this experiment exhibited entrainment, and all seven showed phase-locking to the same narrow range of phase. Results are shown in Figure 10. The mean number of strides before phase locking was 53 (106 steps) occupying more than one minute. The phase ϕ_P_ at which the perturbation torque pulses occurred was assessed for all strides after the onset of phase-locking. The mean was 50.7%, and the standard deviation was 4.00%; a histogram is shown in Figure 8 A. The distribution of ϕ_P_ for experiment 2 was compared with the distribution for the 11 subjects who only performed experiment 1 and exhibited entrainment. An F-test assessed whether ϕ_P_ of these populations came from normal distributions with the same variance; a t-test assessed whether ϕ_P_ of the two populations had the same mean when the standard deviations were assumed equal. Both hypotheses were accepted.

### Role of Auditory Feedback

To test whether entrainment and phase-locking were due to auditory feedback, 6 of the 7 subjects who participated in experiment 2 wore noise-cancelling headphones to mask auditory inputs during both experiments 1 and 2. Entrainment was identified in one or more trials for all 6 subjects, and the mean and standard deviation of ϕ_P_ in experiment 2 were 50.7% and 4.14% respectively (Figure 8 B), statistically indistinguishable from experiment 1.

### Role of Voluntary Intervention

To test whether entrainment and phase-locking were due to voluntary intervention, four of the 6 subjects were instructed to perform a distracting task, counting backward from 100 to 1 in their second language during both experiments 1 and 2. Entrainment was identified in one or more trials for all four subjects, and the mean and standard deviation of the distribution of ϕ_P_ in experiment 2 were 49.8% and 4.13% respectively (Figure 8 C), statistically indistinguishable from experiment 1.

## Discussion

In this study we sought direct evidence to (1) test whether a neuro-mechanical oscillator contributes to level walking and (2) assess the strength of its contribution. Our results suggest that human locomotor control is *not* organized as in reaching to meet a predominantly kinematic specification. Instead, its architecture seems to resemble the supervisory control successfully applied in robotic space exploration to deal with long communication delays.

### Evidence of a Nonlinear Neuro-Mechanical Oscillator

First, robustly sustained oscillation (what von Holst referred to as *Beharrungstendenz*) requires nonlinear dynamics; nonlinear oscillators serve as competent models of biological systems exhibiting rhythmic motion including CPGs [29], [30], [31], [43]. Second, entrainment (what von Holst referred to as *Magneteffekt*) is a distinctive characteristic of nonlinear oscillators [43]. Entrainment was first reported by Huygens in 1665 as an interaction between the periods of two clocks, but a closely related phenomenon can be observed as the response to a strictly periodic perturbation, and that was the approach we chose [32]. We delivered periodic pulses of plantar-flexion torque while subjects walked at constant speed on a treadmill. This entrained the gait of 18 of 19 subjects. Furthermore, we observed phase-locking such that the perturbation assisted plantar-flexion near the ankle push-off phase. Although indirect evidence has been presented previously, to our knowledge, these observations provide the first direct behavioral evidence that some form of nonlinear neuro-mechanical oscillator participates in human locomotion [22], [23].

To what extent did our experimental apparatus influence subjects' gait? Although differences between treadmill and overground walking have been reported, they are subtle, and many studies have employed treadmills as we did. We used a wearable robot to deliver mechanical perturbation. Anklebot interacts with the leg via a shoe and a knee-brace; weighs 3.6 kg with most of that mass concentrated near the knee; and is highly “back-drivable” with a low intrinsic static friction (less than 1 N-m) opposing ankle motion [36]. In a recent study of 9 chronic stroke survivors who walked overground and on a treadmill with and without Anklebot mounted on the paretic leg, Anklebot had no significant effect on the spatio-temporal patterns of gait, even though that study was sufficiently sensitive to detect greater interlimb symmetry during treadmill walking than overground walking [44].

To what extent did our experimental protocol affect our results? For practical reasons, we applied perturbations at periods 50 ms apart. This necessarily limited the resolution with which we could detect a basin of entrainment and may account for the single exceptional subject for whom entrainment was never observed. To minimize uncontrolled variables, all measurements in a single experimental session were made at a constant treadmill speed selected by the subject. Entrainment typically required stride duration to change to synchronize with the periodic perturbation. As treadmill speed did not change, this required a compensatory change of stride length. Consequently, anatomical considerations (e.g. leg length) determined an upper limit on the perturbation period to which entrainment might be observed. The narrow basin of entrainment we observed (Table 2) does not seem to reflect these anatomical constraints. We know of no anatomical factor which would determine a lower limit.

Might entrainment to periodic auditory stimuli account for our results? When generating torque pulses, Anklebot made a perceptible periodic sound. Unimpaired humans may spontaneously synchronize their motions with periodic sounds and, indeed, do so for pleasure. However, synchronization to sounds can occur over a wide range of periods—consider the various cadences of fast and slow dancing—while we observed entrainment only over a very narrow range, 6.7% of preferred gait cadence (Table 2). Furthermore, we observed no change when auditory stimuli were masked by white noise played through noise-cancelling headphones. Entrainment to periodic auditory stimuli cannot account for our observations.

Might subjects have adapted voluntarily? If entrainment and synchrony were achieved by conscious action, most normal walking frequencies should have been entrained. In contrast, we observed that the basin of entrainment was narrower than the typical variability of preferred cadence (Table 2). Furthermore, voluntary adaptation of gait would be expected to occur within a small number of steps if conscious action was involved. In contrast, we observed a long, slow convergence to achieve phase-locking, occupying as many as 60 steps or more (Figure 10). Finally, when subjects performed a distractor task, we observed no change in entrainment behavior. Voluntary adaptation is not a plausible explanation for our observations.

We are unable to rule out involuntary adaptation mediated supra-spinally by afferent feedback. Nevertheless, the weight of evidence is consistent with some combination of peripheral neuro-mechanical factors—oscillatory neural networks (e.g. rhythmic CPGs in the spinal cord or elsewhere); afferent sensory feedback; musculo-skeletal dynamics; and the physical environment. It is well-known that several combinations of these factors may exhibit nonlinear limit-cycle behavior. For example, interaction between the inertial and gravitational mechanics of legs and their intermittent impact with the ground produces a nonlinear limit-cycle oscillation sufficient to yield remarkably coordinated walking on a gentle slope with no control whatsoever—the so-called “passive dynamic walkers” [45], [46]. Minimal active control based on ankle actuation triggered by sensory feedback produces a nonlinear limit-cycle sufficient to yield remarkably coordinated walking on level ground [47]. Of course, spinal neural networks might also produce sustained limit-cycle oscillations and spinal neural networks are also strongly influenced by afferent sensory feedback [21], [24], [48]. Entrainment due to interaction with a peripheral neuro-mechanical oscillator would be consistent with the observations of rhythmic leg movements in response to vibratory or electrical stimulation [22], [23].

Further investigation is needed to reveal the mechanism of entrainment. For example, the periodic perturbation might mechanically assist the musculo-skeletal periphery to be entrained; the robust phase-locking at ankle actuation phase, which is mechanically assistive, is consistent with this hypothesis. Alternatively, it is possible that a neural oscillatory circuit exists and periodic sensory input that is evoked by the mechanical perturbation entrains the neural circuit executing the walking pattern. Any form of the neural oscillatory circuit, including chained reflexes or an internal clock, may entrain and exhibit the observed behaviors.

### Contribution of the Neuro-Mechanical Oscillator

How prominently does a peripheral neuro-mechanical limit-cycle oscillator contribute to human walking? Our observations—the narrow basin of entrainment, the slow phase-locking, and the variability of the after-effect indicate at most a modest contribution.

The narrow basin of entrainment we observed may be due to the modest mechanical perturbation we applied—about 10% of typical maximum ankle push-off torque was applied for a tenth of a second, less than 10% of typical stride duration. A related and equally-plausible interpretation is that a narrow basin of entrainment may be due to a weak coupling between a neuro-mechanical oscillator and the mechanical perturbation we applied. Alternatively, a narrow basin of entrainment may indicate a weakly attracting nonlinear oscillator.

Results from the transient phase dynamics experiment provide corroborating evidence. If the phase of the neuro-mechanical oscillator was strongly attractive, we might expect a rapid convergence to phase-locking. In fact, when the perturbation was initiated at a phase far from its final converged value, 60 or more (sometimes over 100) steps were required to achieve phase locking, occupying a duration of minutes or more. This indicates either a weak attractor or a weak coupling between the neuro-mechanical oscillator and the mechanical perturbation.

However, the variability of the after-effect of the perturbation provides evidence of a weak attractor rather than a weak coupling. In 18 of 19 subjects, exposure to periodic mechanical perturbation was sufficient to evoke entrainment, adaptation of the subject's cadence to match the perturbation period. Adaptation of upper-extremity motions to mechanical perturbations such as Coriolis forces typically evokes a brief after-effect when the perturbation is discontinued, followed by rapid re-adaptation to pre-perturbation behavior [7]. Remarkably, after-effects of the periodic ankle torque perturbation, and re-adaptation when it ceased, were quite variable: 7 of 18 subjects exhibited no after-effect; the subject's cadence returned to its pre-perturbation value within 15 strides. In 2 subjects, within 15 strides after perturbation, the subject's cadence, though statistically different from its pre-perturbation value, was also statistically different from its entrained value; it had begun to “drift” back to its pre-perturbation value (Figure 9 B). However, in 9 subjects, the adapted cadence persisted unchanged for at least 15 strides—30 steps—after the perturbation was discontinued (Figure 6 and 9 A). A strongly attracting limit-cycle oscillator, whether it is coupled strongly or weakly, would be expected to exhibit re-adaptation within a few cycles once decoupled. The variable re-adaptation we observed appears to be consistent with a weakly attractive limit cycle rather than a weak coupling between the oscillators.

Summarizing, the weight of evidence presently available suggests that the neuro-mechanical oscillator is weakly attractive. Though its presence may be detected unambiguously (e.g. by testing for entrainment to periodic mechanical perturbations as in this study) its contribution to locomotor control may be modest. Further study is required to verify these speculations.

### Supervisory Control of Locomotion

Our observations show clear evidence that human locomotor control exhibits distinctive features of a nonlinear oscillator, and that a neuro-mechanical oscillator contributes to human walking. However, it is equally clear that much more is required. Like many mammals, humans can control foot placement with precision while walking, even onto irregularly-spaced footholds. A low-level limit-cycle oscillator cannot account for this behavior. Participation from higher levels of the CNS is indicated, especially if the target footholds are visually acquired. Perhaps the neuro-mechanical oscillator is a legacy of more primitive forms of control. If not, how might these different neural processes interact? Our results suggest the hypothesis that motor control of locomotion in humans is hierarchically organized to implement *episodic supervisory control of a semi-autonomous periphery*.

Supervisory control is proven engineering technology. It was introduced to minimize the computational burden of control and mitigate detrimental effects of time delays due to remote (tele-)operation [49], [50]. It has been especially important in robotic space exploration [51]. Applied to human motor control, the key idea is that, because of the limited response speed of muscles and the substantial delays due to neural conduction, in effect *the supra-spinal nervous system tele-operates the neuro-mechanical periphery*.

The essence of supervisory control is that the “control operator” (the supra-spinal nervous system) has the *option* to intervene directly in the detailed control of “low-level” system behavior (the spinal neuro-mechanical periphery) but—importantly—need not do so continuously [49], [50]. Instead, it only intervenes when need arises (e.g. to react to a stumble or place a foot on a target). In this hypothesis, the neuro-mechanical periphery is conceived to be *semi-autonomous*: it is capable of robustly stable rhythmic walking with minimal central intervention. That requires a nonlinear oscillator because robustly sustained autonomous oscillation can only result from a nonlinear dynamical system. In consequence, the neuro-mechanical periphery would exhibit behavior characteristic of limit-cycle oscillations, including a tendency to entrain to periodic perturbations and converge to a constant phase-locked relation with them, just as we observed.

A modest contribution of the neuro-mechanical periphery to human walking, which is also supported by our observations, provides further support for the proposed supervisory control architecture. The narrow basin of entrainment implies that the semi-autonomous periphery may be entrained to periodic perturbation, but only when the perturbation frequency is close to the original preferred walking cadence. The slow convergence of phase-locking implies that many cycles are required for the external perturbation to entrain the original walking pattern. In sum, the modest contribution of the neuro-mechanical periphery is consistent with its accessibility from the higher levels of the CNS, which consequently would *supervise* the semi-autonomous periphery and easily adjust walking regardless of external perturbations to the periphery.

Supervisory control is necessarily hierarchical. It assumes at least two levels of organization, consistent with the anatomical organization of the CNS. Hierarchical organization of the CNS is by no means a new idea; however, supervisory control requires more than a hierarchical organization. It requires intermittent or episodic communication from the higher level(s) to the lower level(s). Without intermittent communication, communication delays would severely compromise achievable system performance. Episodic supervisory control is a plausible compromise that allows the neuro-mechanical periphery to operate semi-autonomously to unburden the supra-spinal nervous system, yet reserves the option of central intervention as needed to offset the limitations of a semi-autonomous periphery. We suggest that episodic supervisory control may provide a useful perspective to organize some of the vast literature on mammalian locomotion.
